# Geriatrische Dermatologie

**DOI:** 10.1007/s00391-021-02006-2

**Published:** 2022-01-06

**Authors:** Marie Isolde Joura, Kamilla Koszorú, Dóra Czintner, Miklós Sárdy

**Affiliations:** Klinik für Dermatologie, Venerologie und Dermatoonkologie, Semmelweis Universität Budapest, Mária utca 41, 1085 Budapest, Ungarn

**Keywords:** Hautatrophie, Hautalterung, Kontaktekzem, Pruritus, Chronische Wunden, Skin atrophy, Skin ageing, Contact dermatitis, Pruritus, Chronic wounds

## Abstract

**Hintergrund:**

Die Bevölkerung erreicht ein höheres Lebensalter. Begleitend steigt die Inzidenz der Hauterkrankungen.

**Ziel der Arbeit:**

Dargestellt werden die wichtigsten Hauterkrankungen geriatrischer Patienten.

**Material und Methoden:**

Es erfolgten sowohl eine Literaturrecherche in der Datenbank von *PubMed* als auch aus dermatologischen Standardlehrbüchern.

**Ergebnisse:**

Die Haut geriatrischer Patienten reagiert empfindlicher auf Umwelteinflüsse und kann im Rahmen von internistischen Grunderkrankungen mitbetroffen sein. Aufgrund von verzögerter Diagnostik werden maligne Hauterkrankungen bei alten Patienten erst in höheren Stadien diagnostiziert.

**Diskussion:**

Physiologische Hautveränderungen im Alter sind durch entsprechende Pflegemaßnahmen zu behandeln. Bei unklaren Hautveränderungen ist eine rasche dermatologische Abklärung anzustreben.

## Abstract (en)

**Background:**

With an ageing society the incidences of skin diseases increase.

**Objective:**

The most important skin diseases in geriatric patients are discussed.

**Material and methods:**

A literature search was conducted using the PubMed database and standard dermatological textbooks.

**Results:**

Skin diseases in geriatric patients are often more susceptible to external influences and can be affected by visceral diseases. Due to a delayed diagnosis, malignant skin diseases in geriatric patients are first diagnosed at a higher stage.

**Conclusion:**

Physiological skin changes are to be treated with appropriate care. In the case of unclear skin changes, a timely dermatological check-up is to be done.

## Einleitung

Das Fachgebiet der geriatrischen Dermatologie ist aufgrund der steigenden Lebenserwartung von immer größerer Bedeutung. Für die richtige Diagnose und anschließend adäquate Therapie ist das Verständnis der Veränderungen an der Haut sowie der einerseits physiologisch, andererseits durch Umwelteinflüsse und im fortgeschrittenen Alter oft gehäuft auftretenden internistischen Komorbiditäten von großer Bedeutung. Dieser Artikel gibt eine Übersicht über die wichtigsten geriatrischen Hauterkrankungen.

## Die Hautalterung

Der Hautalterungsprozess wird im Laufe des Lebens durch intrinsische und extrinsische Faktoren beeinflusst. Neben ästhetischen Problemen führt die Hautalterung zu einem Funktionsverlust der Haut. Im Allgemeinen erscheint die Altershaut atrophisch, trocken (durch erhöhten Wasserverlust) und rissig. Vermehrt entstehen Barriereschäden. Vorgänge der sich verändernden Haut, wie steigende Anzahl an Mutationen der mitochondrialen DNA und Verkürzung der Telomere, beeinflussen das Altern der Zellen. Genetische Faktoren und oxidativer Stress im Laufe des Lebens sind ebenso Einflussfaktoren für eine epidermale Atrophie. Durch eine sinkende Anzahl an Melanozyten verringert sich der Pigmentgehalt der Haut, unter dem der UV-Schutz leidet. Die Abnahme der Zugfestigkeit, sowie der Elastizität, ist der Degeneration von Kollagen- und elastischen Fasern zu verschulden. Auch Schweiß- und Talgproduktion sind vermindert. Die exogene Hautalterung wird von der UV-Strahlung dominiert, und solare Lentigines sowie aktinische Keratosen bilden sich. Im schlimmsten Fall kann es zu einer Fotokanzerogenese führen. Auch das Rauchen von Tabak ist ein sehr wichtiger Hautalterungsfaktor. Es führt zur Freisetzung von freien Radikalen, des Weiteren degenerieren Kollagen- und elastische Fasern, und tiefe Falten entstehen [19].

## Ekzeme

Aufgrund struktureller und funktioneller Veränderungen der Haut wird sie verwundbarer und wegen des verringerten Wasser- und Fettgehalts der Epidermis trockener. Das daraus resultierende Jucken der Haut ist ein störendes Symptom für die Patienten [34]. Ekzeme können anhand ihrer Pathogenese (z. B. Kontaktekzem, atopische Dermatitis), ihrer Lokalisation (z. B. Handekzem, Analekzem) oder ihrer Aktualität (akutes bzw. chronisches Ekzem) eingeteilt werden.

**Figure 1:**
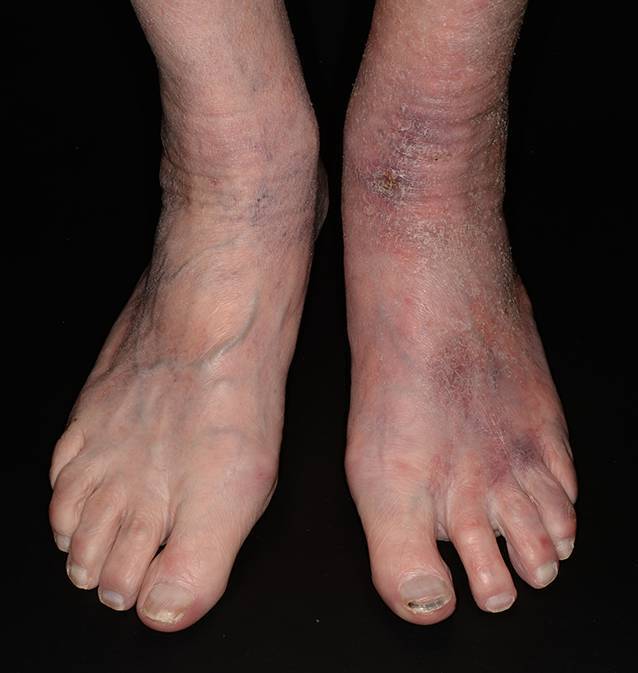

Kontaktekzeme, sowohl allergisch als auch toxisch-kumulativ, verursachen häufig Beschwerden. Der Verlust der epidermalen Hautbarriere und wiederholte Allergenexpositionen (z. B. Nickel) begünstigen die Entwicklung eines allergischen Kontaktekzems. Das auslösende Allergen wird mittels Epikutantest diagnostiziert. Die zwei wichtigsten kumulativ-toxischen Kontaktekzeme sind das Exsikkationsekzem (aufgrund physiologischer Veränderungen alternder Haut, wie z. B. erhöhter Wasserverlust; Abb. 1) und die Inkontinenzdermatitis (durch längere direkte Einwirkung von Stuhl oder Urin auf die Haut im Intimbereich). Bei einem akuten Kontaktekzem erscheinen auf der Haut entzündete, ödematöse, rote Plaques. Im Falle eines chronischen Ekzems können zusätzlich Bläschen, Krusten und Schuppung auftreten. Die juckenden, rissigen, schuppenden und erythematösen Stellen eines Exsikkationsekzems befinden sich gehäuft an den Streckseiten der Extremitäten. Die Therapie besteht aus der Vermeidung der auslösenden Noxen, der topischen Gabe von Glukokortikoiden sowie einer hydratisierenden und rückfettenden topischen Therapie, um die Hautbarrierefunktion wiederherzustellen [26]. Bei einer Inkontinenzdermatitis treten aufgrund der Anatomie häufig Bakterien- und Pilzinfektionen auf und sollten durch pflegerische Maßnahmen verhindert werden [5].

Auch eine atopische Dermatitis darf im hohen Alter nicht außer Acht gelassen werden („late onset“). Etwa 2–3 % der geriatrischen Patienten leiden darunter, verursacht durch Veränderungen des Immunsystems sowie eine herabgesetzte Hautbarrierefunktion. Der starke Pruritus, die verletzte Haut und wiederkehrende *Staphylococcus-aureus*-Infektionen bereiten Beschwerden. Die lichenifizierten Ekzeme erscheinen bevorzugt in den Ellenbeugen und Kniekehlen. Die Diagnose erweist sich aufgrund der vielen Komorbiditäten und diversen Medikamente, welche Pruritus auslösen können, als schwieriger als im jungen Alter [35].

Das nummuläre Ekzem entsteht polyätiologisch und ist charakterisiert durch münzförmig, scharf begrenzte, gelegentlich ödematöse Herde, mit Papulovesikeln, v. a. an den Extremitäten. Die Therapie beginnt mit der Suche nach Fokalinfektionen und richtet sich danach [6].

## Pruritus

Eines der häufigsten Symptome älterer Patienten ist der Juckreiz. Es betrifft knapp 14 % der deutschen Allgemeinbevölkerung [30]. Der häufigste Grund ist eine Xerodermie, eine Folge des physiologischen Hautalterungsprozesses, welche meist schon mit einer Änderung der Hautpflegegewohnheiten behandelt werden kann. Juckreiz kann auch eine Medikamentenreaktion darstellen. Außerdem können dermatologische Krankheiten, wie z. B. Skabies, verschiedene Ekzeme, Urtikaria, Psoriasis, kutane T‑Zell-Lymphome oder bullöse Autoimmundermatosen einen Juckreiz verursachen. Auch diverse andere internistische Grunderkrankungen, wie Diabetes mellitus, Cholestase, Urämie, Hypo- bzw. Hyperthyreose, aber auch maligne Neoplasien oder ein M. Hodgkin können auslösend sein. Gelegentlich sind psychosomatische oder psychiatrische Gründe der Auslöser eines Juckreizes. Des Weiteren kann der Juckreiz auch als paraneoplastisches Syndrom bei soliden Tumoren, Lymphomen oder Leukämien auftreten. Bei einem chronischen Pruritus bestehen die Symptome > 6 Wochen. Es stellt eine interdisziplinäre Herausforderung bezüglich Diagnose und Therapie dar. Eine gründliche Anamnese sowie die klinischen Prurigocharakteristika sind differenzialdiagnostisch wichtig. Das wesentliche Ziel der symptomatischen Behandlung ist die Verbesserung der subjektiven Empfindung, um so die Lebensqualität des Patienten zu verbessern. Primär müssen auslösende Faktoren, wie Grunderkrankungen oder Medikamente, vermieden werden. Eine rückfettende und hydratisierende Hautpflege ist empfohlen, und nichtsedierende systemische H_1_-Antihistaminika können gegeben werden. Erosive Kratzläsionen können topisch mit Antiseptika oder Steroiden behandelt werden [10].

## Chronische Wunden

Das erhöhte Alter, begleitet von diversen Grunderkrankungen, kann Ursache für eine Störung des physiologischen Wundheilungsprozesses sein. Im Alter steigen die Zahlen der Gefäßerkrankungen und des Diabetes mellitus. Somit steigt die Wahrscheinlichkeit für chronische Wunden und die damit verbundenen Komplikationen, begleitet von hohen Kosten für die Behandlungen und einer schlechteren Lebensqualität.

**Figure 2:**
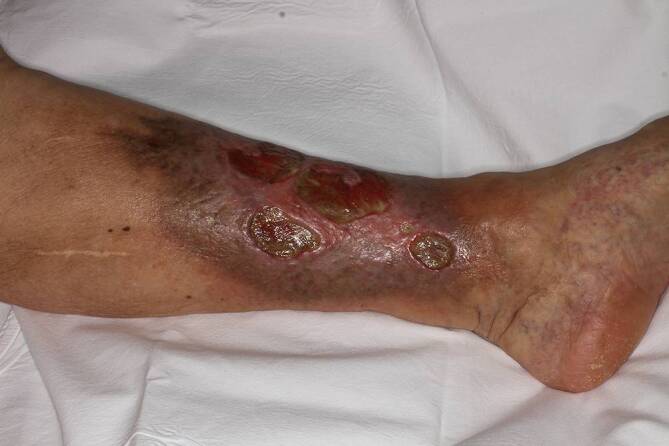

Auf eine chronische Veneninsuffizienz (CVI) ist der größte Teil der Beingeschwüre zurückzuführen. Meist entsteht die Insuffizienz postthrombotisch. Das dermatologische Bild besteht anfangs aus einer Corona phlebectatica am Plantarrand und einem ödematösen Knöchelbereich. Darauffolgend können Hyper- und Depigmentierungen, eine Atrophie blanche, Stauungsekzeme oder trophische Störungen mit Indurationen (Abb. 2) entstehen. Auch Störungen des Nagelwachstums mit Onychomykosen sind möglich [32]. Therapiert werden kann die CVI mittels Bandagen bzw. Kompressionsstrümpfen, um den venösen Rückfluss zu unterstützen. Zusätzlich kann/können eine antiseptische Therapie oder Glukokortikoide angewendet werden. Das nicht allzu schmerzhafte Ulcus cruris stellt die häufigste Komplikation dar, welches meist am distalen Unterschenkel, im Bereich des medialen Knöchels, entsteht [7].

Neben dem Ulcus cruris venosum stellt das Ulcus arteriosum eine wichtige chronische Wunde dar. Lokalisiert an den Extremitäten aufgrund eines Verschlusses der terminalen Arterien, ist es im Gegensatz zum venösen Geschwür schmerzhaft. Die Wundumgebung weist trophische Störungen der Haut und der Hautanhangsgebilde auf. Neben einem abgeschwächten oder fehlenden Puls sowie einer Zyanose ist die kapilläre Auffüllzeit verzögert. Der Patient versucht, durch ein Hochlagern des betroffenen Beines die Schmerzen zu mindern. Eine Angiographie mit anschließender Wiederherstellung der Blutversorgung ist nötig. Bei älteren Patienten sind die Geschwüre oft gemischten Ursprungs [7].

Das diabetische Fußulkus ist meist plantar, am Hallux, an den Os metatarsale I/II/V oder an der Ferse aufzufinden. Es ist mit einer diabetischen Polyneuropathie assoziiert, und somit ist das tiefe Ulkus von milden bis gar keinem Schmerzempfinden begleitet [32].

Ein Dekubitus muss als gravierende Diagnose ernst genommen werden. Oftmals entsteht eine Ulzeration durch eine zu lange Immobilisation, aber auch aufgrund einer Durchblutungsstörung oder sensorischer Defizite. Prädilektionsstellen sind das Sakrum, der Tuber ischiadicum, der Trochanter major femoris und die lateralen Knöchel. Daher ist auf eine korrekte Lagerung von bettlägerigen Patienten zu achten [7].

## Infektionen

Geriatrische Patienten sind aufgrund veränderter Immunität und meist verminderter Körperhygiene anfälliger für Virus- (z. B. Herpes zoster), Bakterien- (z. B. Erysipel) und Pilzinfektionen (z. B. Onychomykose). Die Infektionen verlaufen im Alter oft schwerwiegender und sind mit einer erhöhten Hospitalisations- und Mortalitätsrate verbunden.

Infolge reduzierter Immunität kann es zu einer Reaktivierung des in sensorischen Ganglien gelagerten Varicella-zoster-Virus kommen und somit ein Herpeszoster entstehen. Das betroffene Dermatom (in der Regel nur ein Dermatom auf einer Körperhälfte, ausgenommen Zoster generalisatus) weist, häufig begleitet von starken Schmerzen, ein gürtelförmiges, hellrotes Erythem und ein Ödem mit Vesikeln auf. In ca. 80 % der Fälle geht den Hautmanifestationen ein Prodromalstadium voraus, wobei häufig Fehldiagnosen aufgrund des lokalisationsabhängigen Schmerzes (z. B. Herzinfarkt oder Cholezystitis) gestellt werden. Therapiert wird mit Aciclovir p.o, i.v. bei schwerem Krankheitsbild [9, 12]. Präventiv wird die nachgewiesen effiziente Impfung für Personen ≥ 60 Jahren (bzw. ≥ 50 Jahren bei Grunderkrankung) von der Ständigen Impfkommission (STIKO) empfohlen [2, 22].

Vor allem durch die im erhöhten Alter herabgesetzte zelluläre Immunantwort kommen bakterielle Infektionen gehäuft vor. Wichtig zu erwähnen ist das Erysipel, das meist durch *Streptococcus pyogenes*, welcher über Mikroläsionen in die Haut eindringt (v. a. Beine, aber auch Arme und Gesicht), verursacht wird. Die Entzündung der Dermis und Subkutis spiegelt sich als zungenförmiges Erythem wider, kombiniert mit einem Spannungsgefühl und Druckschmerz. Begleitend kommt es zu Fieber und erhöhten Entzündungsparametern. Erysipelas vesiculosum et bullosum, die nekrotische Variante Erysipelas gangraenosum oder das Erysipelas phlegmonosum stellen schwere Formen dar. Mögliche Komplikationen sind eine Sepsis oder eine tiefe Beinvenenthrombose. Penicillin ist die Therapie erster Wahl. Zusätzlich ist die Epithelverletzung (Eintrittspforte des Bakteriums) zu versorgen [9].

Die sekundäre Infektion einer Intertrigo ist die häufigste Infektion von übergewichtigen geriatrischen Patienten, die oft im Zusammenhang mit einer verminderten Hygiene oder einem Diabetes mellitus steht. Prädilektionsstellen sind aufgrund der anhaltenden Wärme und Feuchte die Achselhöhlen, die Bauchfalten, die Brustfalten und die Leistenregion. Meist erfolgt die Infektion mit *Candida*. Der Patient berichtet über ein brennendes Gefühl und Juckreiz der scharf umschriebenen, erythematösen Plaque. Ein bräunlich, rotbräunliches Bild liegt bei einer Infektion durch das *Corynebacterium minutissimum* vor (Erythrasma). Primär sind eine regelmäßige Reinigung und die Trockenhaltung der prädestinierten Stellen anzustreben. Parallel müssen ein möglicherweise vorhandener Diabetes mellitus therapiert und das Übergewicht reduziert werden. Topisch können antimykotische bzw. antibiotische Arzneimittel verordnet werden. Symptomatisch kann eine milde Glukokortikoidcreme (z. B. Hydrocortison) angewendet werden [28].

Eine in der Geriatrie häufig vorkommende Pilzinfektion ist die Onychomykose. Die Prävalenz steigt durch Risikofaktoren wie Diabetes mellitus, Angio- und Polyneuropathien, Immunsuppression, rezidivierende Traumen und Rauchen. Klinisch ist der Nagel gelblich verfärbt, verdickt oder brüchig. Neben einer topischen Therapie mit Efinoconazol, Tavaborol, Ciclopirox oder Amorolfin ist auch eine systemische Therapie p.o. mit Terbinafin oder Itraconazol möglich, jedoch sind Wechselwirkungen mit der Therapie für die Komorbiditäten möglich. Bei einer Therapieresistenz ist evtl. an eine Laserbehandlung zu denken [13].

**Figure 3:**
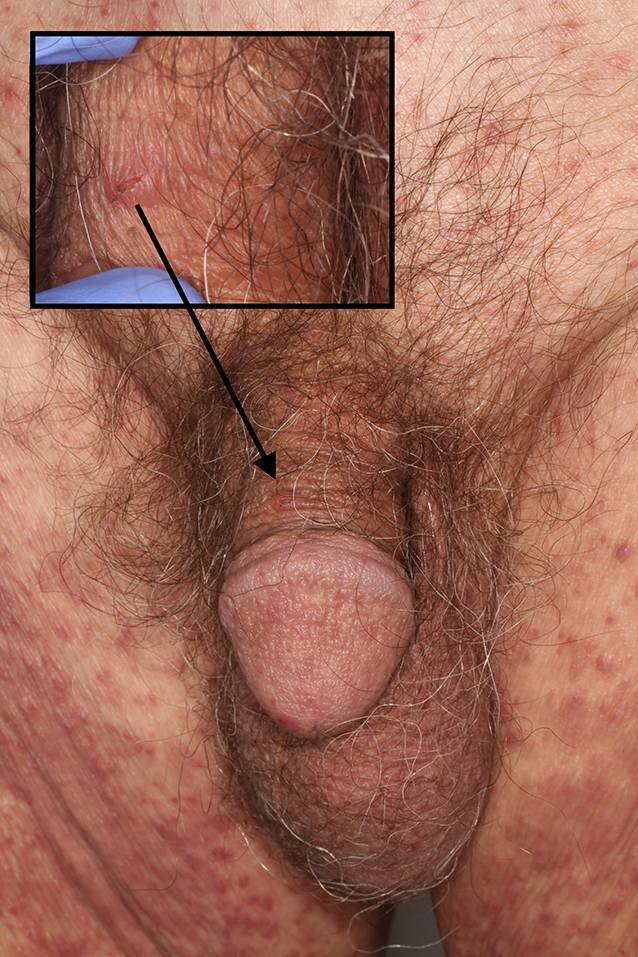

Skabies (Abb. 3) ist eine wichtige Differenzialdiagnose des senilen Juckreizes und bedarf einer schnellen Behandlung. Das Hauptsymptom ist nächtlicher Juckreiz an den charakteristischen Stellen (Fingerzwischenräume, Hände, Handgelenke, Achselhöhlen, Anus, weibliche Areolen und männliche Genitalien). Diagnostisch sind die sichtbaren, gewundenen, einige Millimeter langen Milbengänge. Die Milbe kann als schwarzer Punkt am Ende des Ganges sichtbar sein. Die Therapie erfolgt mit topischem Permethrin oder Ivermectin p.o. Zusätzlich ist auf die körperliche und häusliche Hygiene (v. a. Kleidung und Bettwäsche) zu achten. Die Übertragung auf die im selben Haushalt lebenden Personen ist häufig [33].

## Bullöse Autoimmundermatosen

Autoimmun-bullöse Erkrankungen sind selten, die Inzidenz jedoch steigt mit dem Alter. Das bullöse Pemphigoid (BP) und der Pemphigus vulgaris (PV) sind am häufigsten. Durch die vorhandenen Komorbiditäten der geriatrischen Patienten in Kombination mit gehäuften Medikamentenwechselwirkungen kann es zu einer erschwerten Therapie kommen [16].

Das BP ist die häufigste bullöse Autoimmundermatose. Es bilden sich Autoantikörper gegen Hemidesmosomen basaler Keratinozyten. Folgend kommt es zu einer subepidermalen Blasenbildung, sekundär zu Erosionen, Juckreiz und Eosinophilie. Die Diagnose kann durch das anfangs alleinige Symptom des Juckreizes oder ekzemartige Hautsymptome verzögert sein und somit den Therapiebeginn verzögern [3].

Der PV ist seltener, aber eine schwerwiegendere Erkrankung mit einem früheren Manifestationsalter als das BP. Die Autoantikörper sind gegen Desmoglein gerichtet. Häufig beginnt er mit oralen Mukosaerosionen (Nahrungsaufnahme und Sprechen sind erschwert). Auch können Schleimhäute von Nase, Larynx, Ösophagus, Genitalien oder Anus erkranken. Auf der Haut bilden sich schlaffe Blasen mit klarem Inhalt, die rasch einreißen können und zu Erosionen führen. Über Schmerz wird oft geklagt; der Juckreiz fehlt. Aufgrund von schweren Verläufen und einer schwierigen Therapie kann der PV durch septische Komplikationen tödlich enden [18].

Die Diagnose einer autoimmun-bullösen Hauterkrankung erfolgt anhand des klinischen Bildes, einer histologischen Untersuchung und einer Immunfluoreszenzuntersuchung der Haut sowie des Nachweises zirkulierender Antikörper [4]. Das milde BP (< 10 % Körperoberfläche betroffen) wird primär topisch mit Clobetasolpropionat behandelt. Bei einem schweren Verlauf (> 30 % Körperoberfläche betroffen) ist die Kombination mit einer systemischen Therapie mit Prädnisolonäquivalent empfohlen. Ein milder PV (keine Schmerzen, ≤ 1 % normale Haut oder ≤ 1 cm^2^ Schleimhaut betroffen) wird mit systemischen Kortikosteroiden in Kombination mit einer immunsuppressiven bzw. immunmodulierenden Therapie behandelt. Gegebenenfalls können topisch Kortikosteroide gegeben werden. Anti-CD20-Antiköper, systemische Glukokortikoide und Immunsuppressiva sind bei einem schweren Verlauf indiziert. Eine Wundversorgung sowie eine antiseptische Behandlung zur Vermeidung von bakteriellen Superinfektionen sind empfohlen [18, 29].

## Benigne und maligne Hauttumoren

Mit erhöhtem Lebensalter steigt auch die Wahrscheinlichkeit, an einem benignen oder malignen Tumor zu erkranken. Die zwei großen Gruppen maligner Hauttumoren sind der „weiße Hautkrebs“ (Basalzell- und Plattenepithelkarzinom) und der „schwarze Hautkrebs“ (malignes Melanom).

Die benigne seborrhoische Keratose, die zwar nicht gefährlich ist, aber oftmals ein ästhetisches Problem für die Patienten darstellt, ist im Alter häufig. Die Läsionen sind scharf begrenzt, rund oder oval, sitzen breitbasig auf, haben einen Durchmesser von etwa 0,5–1 cm und befinden sich gehäuft am Rumpf oder am Kopf. Das klinische Bild alleine führt meist zur Diagnose, die durch ein Dermatoskop gesichert werden kann. Die Entfernung aus kosmetologischen Gründen kann durch eine Kürettage mit einem Volkmann-Löffel erfolgen. Die seborrhoische Keratose an sich ist niemals bösartig, kann jedoch beispielsweise als paraneoplastisches Syndrom eines malignen Tumors (Leser-Trélat-Zeichen) auftreten [14].

Ein weiteres ästhetisches Problem können die senilen Hämangiome sein. Sie sind ebenfalls gutartig, wenige Millimeter groß und sattrot. Bei Beschwerden können sie mit einem Laser oder Kauter behandelt werden [8].

Das Keratoakanthom ist aufgrund seines charakteristischen Erscheinungsbildes leicht diagnostizierbar. Es tritt auf sonnenexponierten Stellen auf, besteht aus einem halbkugeligen, hellrot bis rotvioletten Knoten mit einer zentralen Einsenkung, gefüllt von einem keratotischen Pfropf. Aufgrund des schnellen Wachstums ist eine rasche Exzision empfohlen [20].

Eine häufige Präkanzerose ist die aktinische Keratose (AK). Die UV-Strahlung der Sonne ist der wichtigste Risikofaktor. Betroffen sind v. a. chronisch dem Sonnenlicht ausgesetzte Bereiche (Kopfhaut, Ohren, Gesicht, Dekolleté, Arme, Rücken). Aus der AK kann sich nach Jahren ein Plattenepithelkarzinom entwickeln. Es gibt diverse Therapiemöglichkeiten, wie die Kryotherapie, topisches Fluorouracil, Imiquimod, fotodynamische Therapie, chirurgische Exzision oder Lasertherapie. Bei Nichtansprechen auf die Therapie oder untypischem klinischem Bild ist eine histologische Abklärung notwendig [21, 31].

Auch der M. Bowen ist eher eine Erkrankung älterer Patienten. Es handelt sich um ein In-situ-Plattenepithelkarzinom. Typisch ist eine langsam wachsende, psoriasisartige Plaque (meist am Stamm). Die Oberfläche erscheint trocken, schuppig, erythematös, hyperkeratotisch und hat einen zerklüfteten Rand. Die Kryotherapie, die Anwendung von 5‑Fluorouracil, die Kürettage oder die Exzision sind empfohlen, um die Entwicklung eines Bowen-Karzinoms (Plattenepithelkarzinom) zu verhindern [27].

**Figure 4:**
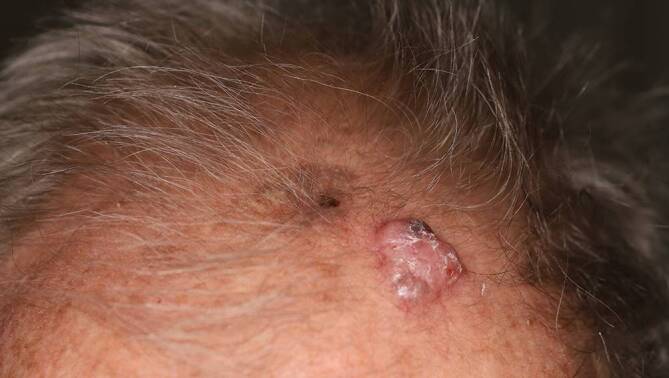

Das Basalzellkarzinom (Abb. 4) ist der am häufigsten auftretende maligne Hauttumor und entsteht de novo. Die Inzidenz steigt mit dem Lebensalter und der Sonnenexposition. Multiple Primärtumoren können beim selben Patienten innerhalb von Jahren bis Jahrzehnten auftreten. Am meisten betroffen ist das Gesicht (primär die Nase), gefolgt von Hals, Rumpf und Extremitäten. Das Erscheinungsbild ist variabel. Der häufigste Subtyp, das noduläre Basalzellkarzinom, ist scharf begrenzt, breitbasig, wachsartig, umgeben von einem perlschnurartigen Randsaum und durchzogen mit Teleangiektasien. Häufig befindet sich zentral eine Einsenkung, die im Laufe der Zeit ulzerieren kann. Die Diagnose wird anhand des klinischen Bildes, unterstützt von einem Dermatoskop und einer histologischen Untersuchung, gestellt [23]. Es metastasiert selten, zeigt jedoch ein lokal destruierendes Wachstum. Deswegen bedarf es einer schnellen Therapie. Die vollständige chirurgische Exzision ist der Standard. Gegebenenfalls können eine Flachexzision, Strahlentherapie, topische Therapie, fotodynamische Therapie, Kryotherapie, Laser oder systemische Therapie angewendet werden. Das Basalzellkarzinom zeigt aber eine hohe Rezidivrate [24].

**Figure 5:**
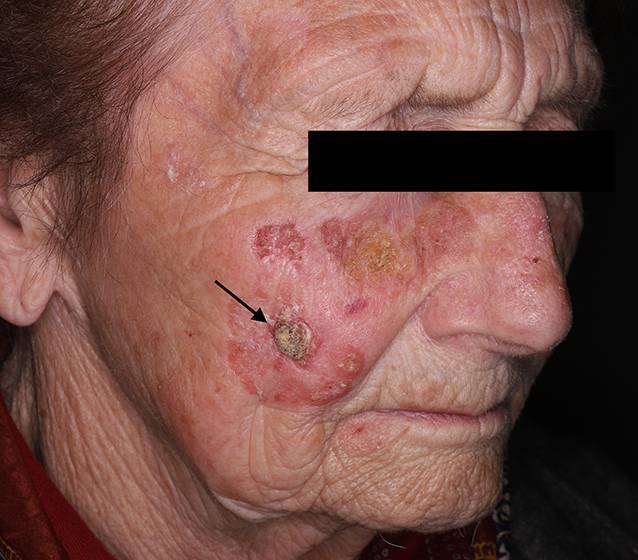

Seltener als das Basalzellkarzinom kommt das Plattenepithelkarzinom (Abb. 5) vor. In den meisten Fällen entwickelt es sich aus atypischen Keratinozyten [15]. Die Ätiologie ist multifaktoriell. Neben den exogenen Faktoren, wie z. B. UV-Strahlung oder chemischen Karzinogenen, ist auch die genetische oder immunologische Prädisposition entscheidend. Die Standardtherapie besteht aus der vollständigen Exzision. Bei einem nicht vollständig resezierbaren Karzinom oder ausgedehnten Lymphknotenbefall, sowie bei inoperablen Patienten, kann eine adjuvante bzw. postoperative Strahlentherapie angewendet werden [25].

**Figure 6:**
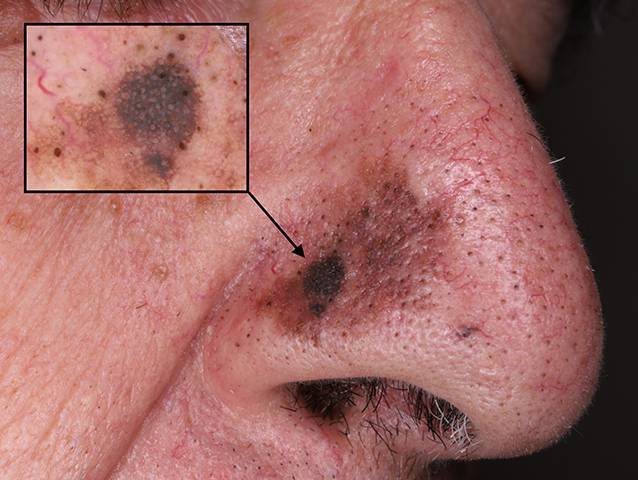

Das maligne Melanom (Abb. 6) ist der häufigste Hauttumor mit letalem Ende. Es ist seltener als ein Basalzell- oder Plattenepithelkarzinom, jedoch zeigt es eine stärkere Aggressivität, welche sich in einer frühen Metastasierung widerspiegelt. Auch hier ist die UV-Strahlung der wichtigste exogene Risikofaktor. Die Tumoren erscheinen meist unregelmäßig begrenzt sowie mehrfarbig [11]. Anhand des klinischen Bildes (ABCDE-Regel: Asymmetrie, Rand, Farbe, Durchmesser, Entwicklung) [1] und einer Histologie wird die Diagnose gestellt. Die Therapie erfolgt anhand einer chirurgischen Exzision des Primärtumors mit entsprechendem Sicherheitsabstand [11]. Bei älteren Patienten werden Melanome sehr häufig erst in fortgeschrittenen Stadien diagnostiziert und behandelt [17].

## Fazit für die Praxis

- Die physiologischen Veränderungen der Haut im Alter können durch konsequente Pflege gemildert werden. Schädliche Einflüsse von außen sind zu minimieren.
- Ein regelmäßiges dermatologisches Screening mit dem Dermatoskop sowie einer Fotodokumentation sollte durchgeführt werden, um maligne Tumoren frühzeitig zu erkennen.
- Bei unklaren Befunden, bei Verdacht auf Malignität, bei Nichtansprechen auf Therapien (spätestens nach 3 Monaten) sowie bei komplexen Hauterkrankungen, die eine fachspezifische Erfahrung benötigen, sollte nicht gezögert werden, ein dermatologisches Konsil anzufordern.
